# Essential Roles of the Kar2/BiP Molecular Chaperone Downstream of the UPR Pathway in *Cryptococcus neoformans*

**DOI:** 10.1371/journal.pone.0058956

**Published:** 2013-03-06

**Authors:** Kwang-Woo Jung, Hyun Ah Kang, Yong-Sun Bahn

**Affiliations:** 1 Department of Biotechnology, Center for Fungal Pathogenesis, Yonsei University, Seoul, Korea; 2 Department of Life Science, Center for Fungal Pathogenesis, College of Natural Science, Chung-Ang University, Seoul, Korea; Research Institute for Children and the Louisiana State University Health Sciences Center, United States of America

## Abstract

The endoplasmic reticulum (ER) is a central hub where secreted or membrane-bound proteins are maturated and folded properly in eukaryotes. Maintenance of ER homeostasis is particularly important for human fungal pathogens, such as *Cryptococcus neoformans*, which encounter a plethora of host-mediated stresses during infection. Our previous study demonstrated that the unfolded protein response (UPR) pathway, composed of the evolutionarily conserved Ire1 kinase and the unique Hxl1 transcription factor, has pleiotropic roles in ER stress response, thermotolerance, antifungal drug resistance, and virulence in *C. neoformans*. Here, we functionally characterized an ER-resident molecular chaperone, Kar2/BiP, in *C. neoformans*. Conditional expression of *KAR2* by the copper-regulated promoter revealed that Kar2 is essential for the viability of *C. neoformans*. Constitutive expression of *KAR2* by the strong histone H3 promoter partially restores resistance to ER stress, cell wall stress, thermotolerance, and genotoxic stress in *ire1*Δ and *hxl1*Δ mutants, suggesting that Kar2 mainly functions downstream of the UPR pathway. Furthermore, Kar2 appears to control azole resistance in *C. neoformans* downstream of the UPR pathway without regulation of *ERG11* or *ERG3*. Interestingly, we discovered that azole treatment is sensed as ER-stress and subsequently activates the Ire1-dependent Hxl1 splicing event and induction of *KAR2* by the UPR pathway. In contrast, the constitutive expression of Kar2 is not sufficient to restore the Ire1-mediated regulation of capsule production in *C. neoformans* UPR mutants. In conclusion, this study demonstrates that Kar2 is not only essential for vegetative growth but also required for response and adaptation to the environmental stresses and antifungal drugs downstream of the UPR pathway in *C. neoformans.*

## Introduction

Sensing, responding, and adapting to environmental cues, such as nutrient starvation, hypoxia, and temperature changes, are essential for all living organisms. To cope with such stresses, all organisms have evolutionarily conserved and unique signal transduction pathways depending on their biological niches. Particularly, those involved in homeostasis of the endoplasmic reticulum (ER) play a crucial role in protein quality control for eukaryotes because secreted or membrane proteins must be assembled or folded properly in the ER prior to their cellular localization. When misfolded or unfolded proteins accumulate beyond the protein folding capacity of the ER due to external environmental cues or internal physiological changes – a condition known as ER stress – the unfolded protein response (UPR) and ubiquitin dependent ER-associated degradation (ERAD) pathways are activated [1], [2].

The molecular mechanism of the UPR pathway is well characterized in budding yeast *Saccharomyces cerevisiae* [3]. Upon exposure to ER stress, the ER transmembrane-resident Ire1 kinase undergoes autophosphorylation and induces spliceosome-independent, unconventional splicing of *HAC1* mRNA, which encodes the downstream transcription factor of Ire1 [4]. The activated Hac1 transcription factor induces diverse UPR target genes, including those involved in translocation, glycosylation/modification, and protein folding and degradation, to alleviate ER stress [5]. The UPR pathway is critical for virulence regulation in opportunistic human fungal pathogens [6]–[9]. In *Aspergillus fumigatus*, which is an ascomycete filamentous fungus causing invasive and systemic aspergillosis, deletion of the Hac1 transcription factor or the Ire1 kinase gene results in severe virulence attenuation [6], [9]. Hac1 regulates the morphology of *Candida albicans*, which is an ascomycete pleomorphic fungus causing superficial, vaginal, and systemic candidiasis, by modulating the expression of genes encoding cell surface proteins [8].

In the basidiomycete fungus *Cryptococcus neoformans*, the UPR pathway also has essential roles in controlling its virulence as well as the ER stress response [7]. Notably, the *Cryptococcus* UPR pathway comprises the evolutionarily conserved Ire1 kinase and the unique bZIP transcription factor, Hxl1, which is phylogenetically divergent from the conventional yeast Hac1 or human Xbp1 proteins. Interestingly, Ire1 appears to have both Hxl1-dependent and -independent functions. Ire1 modulates ER stress response, thermotolerance, maintenance of cell wall integrity, and azole drug resistance in an Hxl1-dependent manner. In contrast, Ire1 also has Hxl1-independent roles in controlling capsule production and certain stress responses. Interestingly, the *hxl1Δ* mutant shows a greater thermosensitivity than the *ire1Δ* mutant, suggesting that an Ire1-independent signaling circuit could partly contribute to Hxl1 regulation and activation [7].

Kar2, also known as BiP, is not only an ER-resident molecular chaperone but also a common negative regulator of the UPR pathway in yeast and animal cells. It is essential for viability and involved in diverse cellular processes, including protein translocation and ER-associated degradation [10]–[14]. As the ER luminal Hsp70 molecular chaperone, *KAR2* is induced in response to heat shock and treatment with tunicamycin, which is an ER stress-inducer inhibiting N-linked glycosylation [15]. Kar2 induced by the UPR pathway alleviates ER stress to interact with misfolded or unfolded proteins as a molecular chaperone. Although *KAR2* mRNA levels are modulated via the UPR pathway, Kar2 itself is an important regulator of the UPR pathway. In the absence of ER stress, Kar2 physically interacts with and inactivates the Ire1 sensor. In response to ER stress, Kar2 dissociates from Ire1, which subsequently dimerizes or oligomerizes for activation [16], [17]. Thereby, Kar2 limits unrestricted activation of the UPR pathway under unstressed conditions.

Our prior study identified a *Cryptococcus* gene (CNAG_06443.2) orthologous to yeast Kar2 and demonstrated that the expression of *KAR2* is controlled by the UPR pathway in *C. neoformans* in response to ER stress and temperature upshifts [7]. Nevertheless, the cellular roles of Kar2 remain elusive in basidiomycete fungi, including *C. neoformans*. In this study, we functionally characterized the *Cryptococcus* Kar2 through the construction and phenotypic analysis of conditional and constitutive *KAR2* overexpression strains. Here, we discovered that Kar2 is not only required for viability, but also as an important chaperone downstream of the Ire1/Hxl1-dependent UPR pathway in *C. neoformans*.

## Results

### Identification of the *KAR2* gene in *C. neoformans*

**Figure 1:**
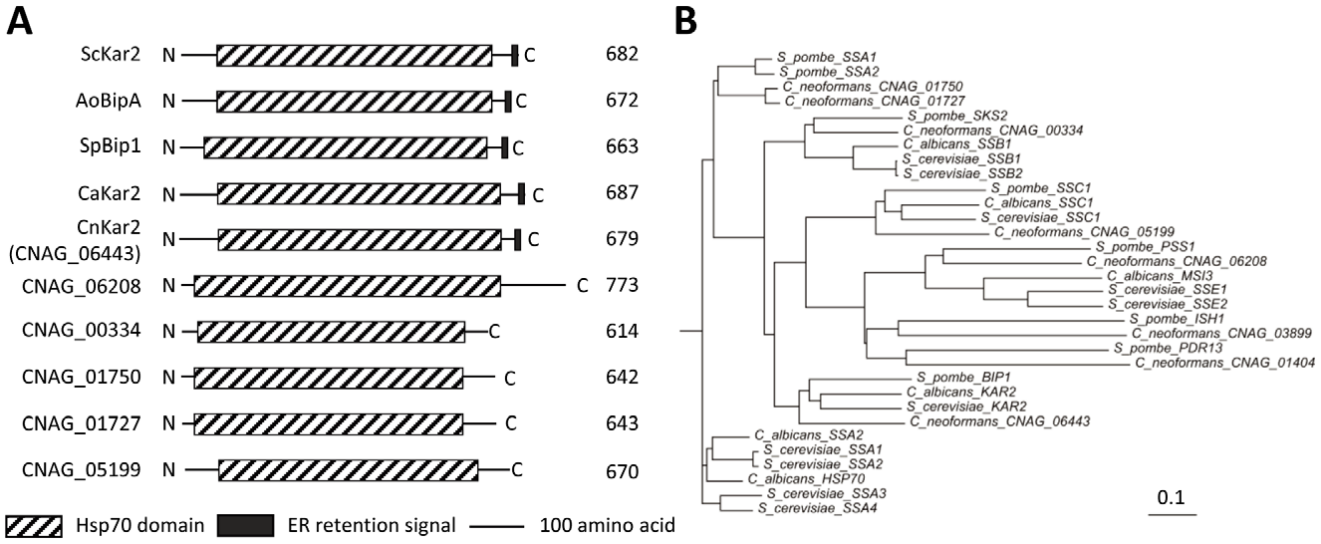
Identification of Kar2/BiP in *C. neoformans*. (A) Schematic outline of Kar2/BiP proteins in fungi and Hsp70 proteins in *C. neoformans*. The box with dashed line indicates an Hsp70 domain. The black box represents an ER retention signaling motif at the C-terminus [HDEL for *S. cerevisiae* Kar2 (ScKar2), *Aspergillus oryzae* BipA (AoBipA), *C. albicans* Kar2 (CaKar2) and *C. neoformans* Kar2 (CnKar2) or ADEL for *Schizosaccharomyces pombe* Bip1 (SpBip1)]. Each number represents the size of the protein in amino acid. (B) Phylogenetic tree of Kar2/BiP proteins and Hsp70 family proteins in *C. neoformans* and in other fungi. The phylogenetic tree was generated by the philodendron phylogenetic tree printer (http://iubio.bio.indiana.edu/treeapp/treeprint-form.html). The scale bar represents the evolutionary distance of 0.1. Each protein sequence used for phylogenetic analysis was retrieved from the following genome database [*Saccharomyces* Genome Database (http://www.yeastgenome.org/), *Candida* Genome Database (http://www.candidagenome.org/), *S. pombe* GeneDB (http://old.genedb.org/genedb/pombe/), and *C. neofomans* var. *grubii*. H99 Database (http://www.broadinstitute.org/annotation/genome/cryptococcus_neoformans/MultiHome.html)].

To identify a *KAR2* ortholog in *C. neoformans*, we performed BLAST searches (blastp) with the *S. cerevisiae* Kar2 protein sequence as a query. In the genome from serotype A H99 strain, several Kar2 orthologous genes were discovered [CNAG_06443.2 (score: 786.563, *e*-value: 0), CNAG_01750.2 (score: 697.197, *e*-value: 0), CNAG_01727.2 (score: 693.345, *e*-value: 0), CNAG_00334.2 (score: 598.201, *e*-value: 0), CNAG_05199.2 (score: 515.383, *e*-value: 0), and CNAG_06208.2 (score: 258.84, *e*-value: 0)]. These may reflect the presence of a diverse Hsp70 class of proteins in *C. neoformans*. The protein domain analysis revealed that CNAG_06443.2 contains an ER retention signal at the C-terminus (HDEL), which is conserved among Kar2/BiP proteins in other fungi (Fig. 1A). The phylogenetic analysis showed that CNAG_06443.2 is evolutionarily more closely related to known Kar2/BiP proteins than to other Hsp70 family proteins (Fig. 1B). Therefore, the gene CNAG_06443.2 was named Kar2/BiP in *C. neoformans*.

### Kar2/BiP is essential for the viability of *C. neoformans*

**Figure 2:**
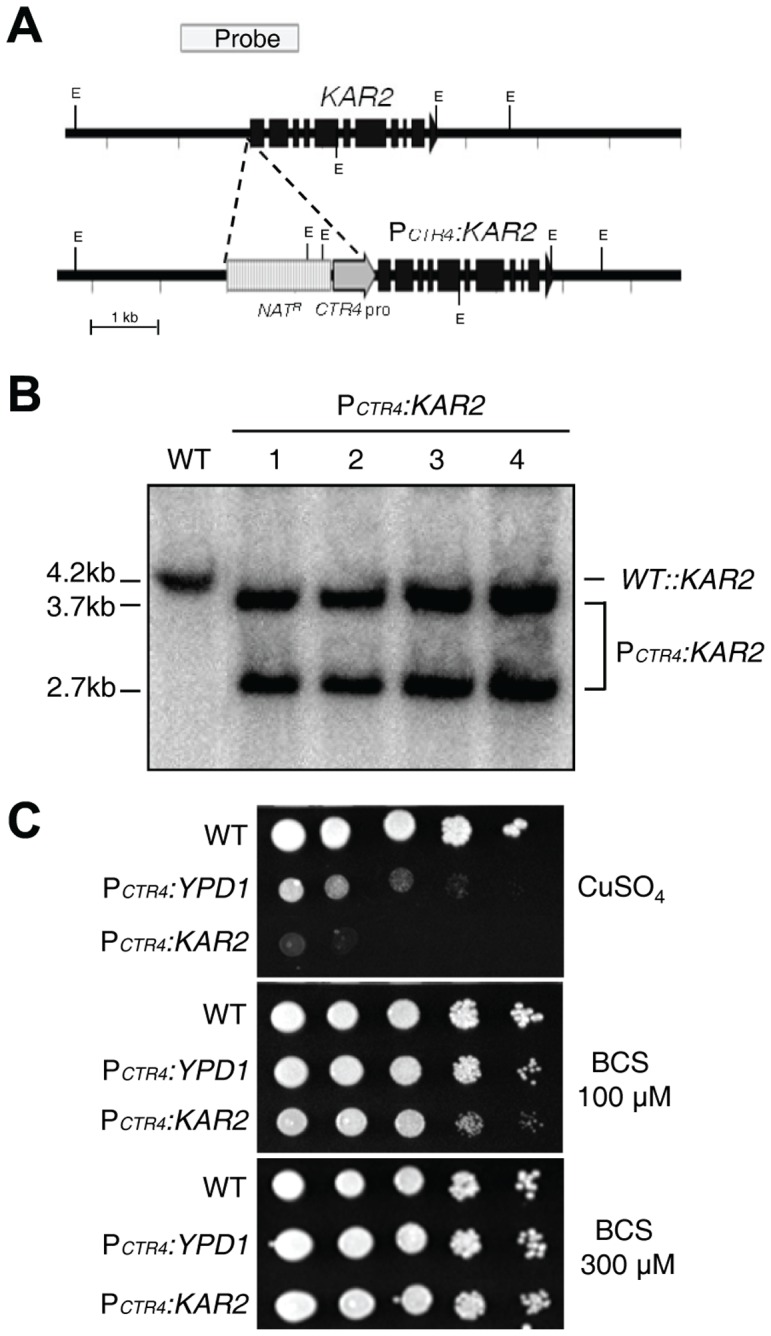
Kar2/BiP is essential for the viability of *C. neoformans*. (A) The scheme for the construction of the P*_CTR4_:KAR2* promoter insertion strain. A construct containing the *CTR4* promoter and the *NAT* dominant selectable marker was inserted 8 bp upstream of the ATG start site of the *KAR2* gene. Black boxes indicate exons of the *KAR2* gene. (B) The correct genotypes of the P*_CTR4_:KAR2* promoter strains [four independent strains (YSB1637, YSB1638, YSB1639, and YSB1640 as labeled 1 to 4, respectively)] compared to the WT H99 strain were confirmed by Southern blot analysis using genomic DNAs digested with the restriction enzyme EcoRΙ. The membrane was hybridized with a *KAR2*-specific probe, washed, and developed. (C) The WT H99, the P*_CTR4_:KAR2* (YSB1637), and P*_CTR4_:YPD1* (YSB859, a positive control) strains were cultured overnight at 30°C in a liquid YPD medium, 10-fold serially diluted, and spotted onto yeast nitrogen base agar (YNB) medium containing 12.5 µM CuSO_4_, 100 µM BCS, or 300 µM BCS. Cells were incubated at 30°C for 4 days and photographed.

Kar2 is known to be essential for the viability of *S. cerevisiae* and *C. albicans*, shown by the construction and analysis of the conditional null mutant [10]–[12]. To address whether Kar2 is also essential for the growth of *C. neoformans*, we constructed conditional null *kar2* mutants by inserting a copper regulated promoter (*CTR4* promoter; P*_CTR4_*) right upstream of the ATG start codon of the *KAR2* gene (Fig. 2A). To verify the exact start codon of the *KAR2* gene, we performed rapid amplification of cDNA ends (RACE) for the 5′ untranslated region (UTR) (GenBank accession number JX982102). After we confirmed the targeted insertion of the *CTR4* promoter right upstream of the *KAR2* gene by Southern blot analysis (Fig. 2B), we measured the growth defect of P*_CTR4_*:*KAR2* strains in a YNB medium containing bathocuproine disulfonate (BCS, a copper chelator), which induces the *CTR4* promoter, or copper sulfate (CuSO_4_), which represses the *CTR4* promoter. The wild-type (WT) strain did not show any growth defects in both BCS and CuSO_4_-containing YNB media, whereas the P*_CTR4_*:*YPD1* strain, which was spotted as a positive control, exhibited a severe growth defect in the YNB+CuSO_4_ medium as previously described [18] (Fig. 2C). The *YPD1* gene encodes a histidine-containing phosphotransfer protein essential for the growth of *C. neoformans* [18]. Interestingly, the P*_CTR4_*:*KAR2* strains displayed greater growth defects than those of the P*_CTR4_*:*YPD1* strain in the YNB+CuSO_4_ medium, whereas their growth defects were suppressed in the YNB+BCS medium (Fig. 2C). To indirectly support these data, repeated trials to construct *kar2*Δ deletion mutants were not successful (data not shown). In conclusion, *KAR2* is required for the viability of *C. neoformans*.

### Construction of *KAR2* overexpression strains

**Figure 3:**
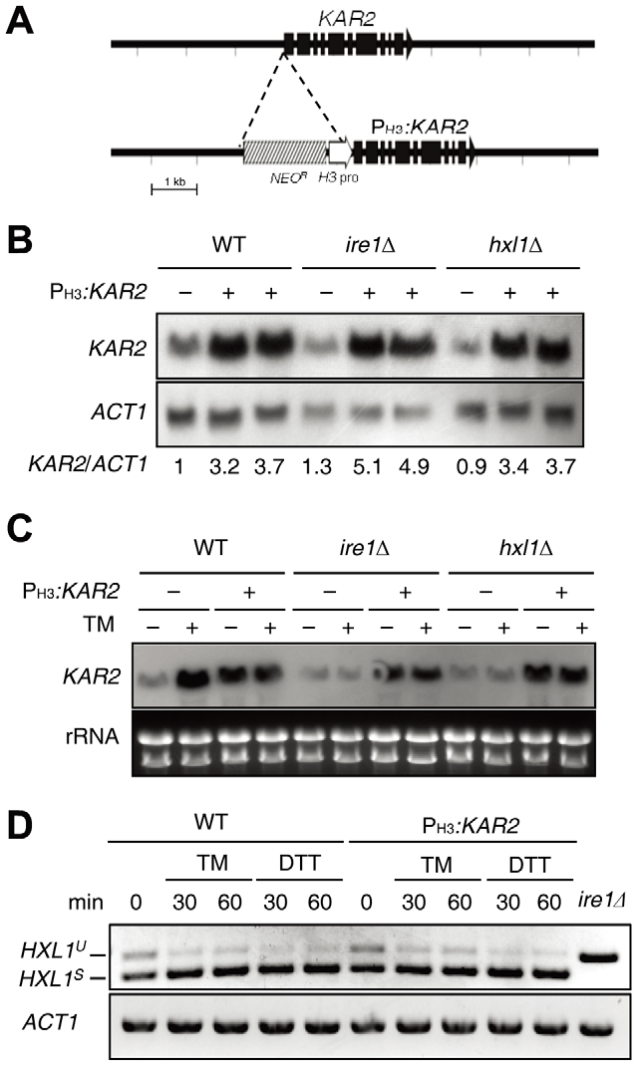
Construction of the constitutive *KAR2* expression strain in *C. neoformans*. (A) The strategy for the construction of the P_H3_ *:KAR2* strain containing the *NEO* resistance marker (*NEO* ^R^) and the histone *H3* gene promoter (*H3* pro). (B) Northern blot analysis for measuring *KAR2* expression in P_H3_ *:KAR2* strains [YSB1751 (lane 2), YSB1752 (lane 3), YSB1741 (lane 5), YSB1744 (lane 6), YSB1745 (lane 8), and YSB1746 (lane 9)] and their parent strains [WT H99 strain (lane 1) and *ire1*Δ (lane 4) and *hxl1*Δ (lane 7) mutants]. *KAR2* expression levels were quantitatively measured with a phosphorimager and normalized to *ACT1* expression levels. Each *KAR2*/*ACT1* is a value relative to that of the WT strain set to 1.0. (C) Northern blot analysis for measuring *KAR2* expression in WT, *ire1*Δ, *hxl1*Δ mutants, and P_H3_ *:KAR2* strains (YSB1751, YSB1741, and YSB1745) treated with or without TM (0.3 μg/ml) for 1 h. (D) RT-PCR analysis of UPR-induced *HXL1* splicing with cDNA samples in WT H99 strain and P_H3_ *:KAR2* strain (YSB1751) treated with or without TM (0.3 μg/ml) or DTT (20 mM). RT-PCR of *HXL1* and *ACT1* was performed with gene-specific primers listed in the Materials and Methods.

Our previous study showed that expression of *KAR2* is induced by tunicamycin (TM), which is an ER stress inducer, or by a temperature upshift (from 30°C to 37°C) in WT, but not in *ire1*Δ and *hxl1*Δ mutants, suggesting that Kar2 is one of the UPR downstream target genes in *C. neoformans* [7]. As described before, however, Ire1 and Hxl1 have both mutually dependent and exclusive roles in *C. neoformans*. Therefore, we next wished to determine which subsets of the Ire1- and Hxl1-dependent phenotypes are controlled by Kar2. For this purpose, we constructed constitutive *KAR2* expression strains in a WT strain and *ire1*Δ and *hxl1*Δ mutant backgrounds by inserting the histone H3 gene promoter right upstream of the ATG start codon in the *KAR2* gene (Fig. 3A). We verified the correct insertion of the P_H3_ *:KAR2* alleles through Southern blot analysis (data not shown) and performed Northern blot analysis to measure the basal expression levels of *KAR2* in the WT, *ire1*Δ, *hxl1*Δ, and P_H3_ *:KAR2* strains (Fig. 3B). As expected, the expression levels of *KAR2* were 3- to 5-fold higher in all P_H3_ *:KAR2* strains in a WT, *ire1Δ*, or *hxl1Δ* strain background (Fig. 3B).

To examine whether *KAR2* in the P_H3_ *:KAR2* strain is constitutively expressed under ER stress, we monitored the expression levels of *KAR2* in response to TM. Supporting previous data, *KAR2* expression was highly induced in response to TM in WT but not in *ire1Δ* and *hxl1Δ* mutants, whereas *KAR2* overexpression levels in the P_H3_ *:KAR2* strain were maintained stably under ER stress (Fig. 3C). Notably, TM-induced *KAR2* expression levels in WT were higher than those in the P_H3_ *:KAR2* strain (Fig. 3C), indicating that the native *KAR2* promoter could be stronger than the H3 promoter under certain ER stressed conditions.

Finally, we examined whether increased basal expression of *KAR2* in the P_H3_ *:KAR2* strain might inhibit activation of the Ire1-mediated unconventional splicing of *HXL1* mRNA, given the known fact that Kar2 serves as a negative regulator of Ire1 in yeast [16], [17]. As previously described [7], both spliced (*HXL1^S^*) and unspliced (*HXL1^U^*) versions of *HXL1* mRNA are observed even under an unstressed condition in *C. neoformans*, but the spliced version is predominant upon treatment with ER stress inducers (Fig. 3D). The basal ratio of *HXL1^S^* and *HXL1^U^* under the unstressed condition and the Ire1-mediated unconventional splicing of *HXL1* by ER stress occurred in a similar fashion in the P_H3_ *:KAR2* strain, indicating that the increased basal expression of *KAR2* by the H3 promoter does not affect basal activation and ER stress-mediated induction of the UPR pathway in *C. neoformans*.

### Kar2 controls ER stress response, high temperature growth, and cell wall integrity downstream of the UPR pathway in *C. neoformans*

**Figure 4:**
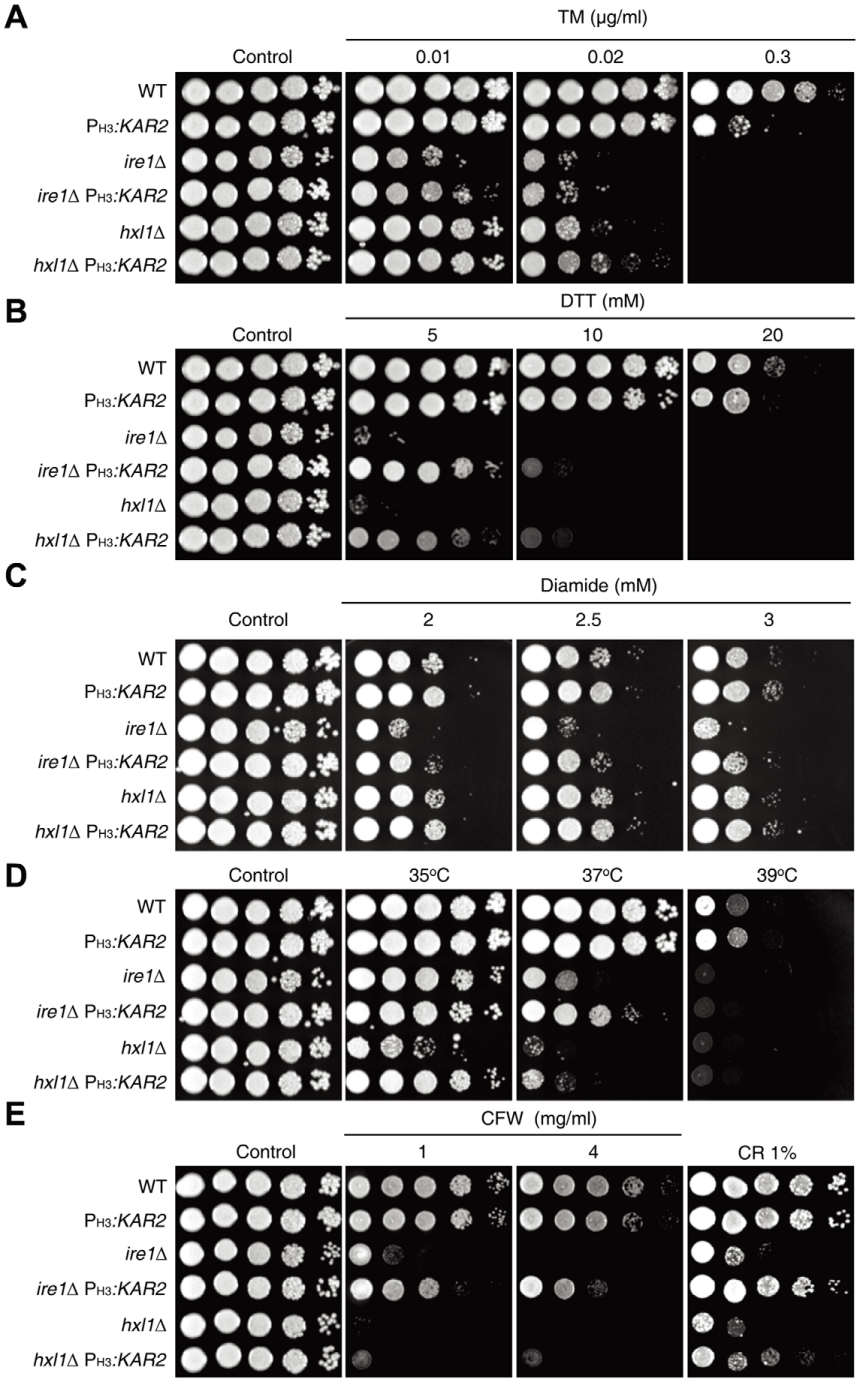
Kar2 is involved in ER stress response, thermotolerance, and maintenance of cell wall integrity downstream of the UPR pathway. The *KAR2* overexpression strains [P_H3_ *:KAR2* (YSB1751), *ire1*Δ P_H3_ *:KAR2* (YSB1741), and *hxl1*Δ P_H3_ *:KAR2* (YSB1745)] and their parent strains (WT H99 strain and *ire1*Δ and *hxl1*Δ mutants) were grown for 16 h at 30^o^C in a liquid YPD medium, 10-fold serially diluted, and spotted on a YPD agar medium containing the indicated concentrations of tunicamycin (TM; A), dithiothreitol (DTT; B), diamide (C), calcofluor white (CFW; E), and congo-red (CR; E). Strains were incubated at 30°C or at 35, 37, or 39°C for thermotolerance test (D) for 3–4 days and were photographed.

To elucidate the functions of Kar2 in ER stress response and adaptation, we examined whether *KAR2* overexpression could suppress the ER stress sensitivity of UPR mutants. Previously, we have reported that the *ire1*Δ and *hxl1*Δ mutants exhibit equal levels of hypersensitivity to high concentrations of 2 ER stress-inducing agents, TM (0.075 µg/ml) and dithiothreitol (DTT; 10 mM). In this study, however, we have found that the *ire1*Δ mutant was more sensitive to TM than the *hxl1*Δ mutant at lower levels of TM (<0.02 µg/ml) (Fig. 4A), indicating that Ire1 may utilize other downstream effectors besides Hxl1 in counteracting ER stress. *KAR2* overexpression partially suppressed the growth defects of *ire1*Δ and *hxl1*Δ mutants in response to low concentrations of TM (<0.02 µg/ml) and DTT (<5 mM) (Fig. 4A and 4B), indicating that Kar2 is one of the key effectors to counteract ER stress downstream of the Ire1/Hxl1-dependent UPR pathway in *C. neoformans*. At high concentrations of TM (>0.02 µg/ml) or DTT (>5 mM), however, the suppressive effect of *KAR2* overexpression in the UPR mutants was not evident (Fig. 4A and 4B), probably because *KAR2* expression levels by the H3 gene promoter might not be sufficient or other multiple UPR downstream effectors are required for a full-scale ER response and adaptation. In fact, *KAR2* expression by the H3 gene promoter appeared to be less efficient in counteracting the ER stresses exerted by the high concentrations of TM (0.3 µg/ml) or DTT (20 mM) than by the native *KAR2* promoter in WT strain (Fig. 4A and 4B).

Notably, the suppressive effect of *KAR2* overexpression in the UPR mutants was more evident under DTT than under TM (Fig. 4A and Fig. 4B). This may explain the molecular chaperonic role of Kar2 for resolving unfolded proteins given that DTT is a reducing agent, which disrupts protein disulfide bonds and normal protein structures to yield unfolded or misfolded proteins. With an action opposite to that of DTT, diamide is a thiol (SH)-specific oxidant, which induces abnormal disulfide bond formation and also perturbs natural protein structures. Our previous study revealed that the *ire1*Δ mutant, but not *hxl1*Δ mutant, exhibits increased diamide sensitivity [7], indicating that Ire1 has an Hxl1-independent manner in regulating diamide resistance. *KAR2* overexpression also recovered diamide-resistance in the *ire1*Δ mutant (Fig. 4C).

Similarly, *KAR2* overexpression was able to partly suppress the temperature-sensitive (TS) growth defect of the *ire1*Δ and *hxl1*Δ mutants. As previously reported [7], the *hxl1*Δ mutant exhibited even greater TS growth defects than the *ire1*Δ mutant compared with WT (Fig. 4D), which is in stark contrast to the case of TM-sensitivity. These data indicate that Hxl1 has an Ire1-independent role in controlling thermotolerance. At 37°C, *KAR2* overexpression significantly restored the normal growth of the *ire1*Δ mutant but only slightly restored the growth of the *hxl1*Δ mutant (Fig. 4D). Growth recovery of the *hxl1*Δ mutant by *KAR2* overexpression was evident at 35°C (Fig. 4D). At 39°C, however, *KAR2* overexpression did not rescue the growth defects of *ire1*Δ and *hxl1*Δ mutants (Fig. 4D).

Related to the ER stress response and thermotolerance, the UPR pathway is required for maintenance of cell wall integrity in *C. neoformans*. Therefore, both *ire1*Δ and *hxl1*Δ mutants are highly sensitive to cell wall destabilizing agents, such as Congo red (CR) and calcofluor white (CFW). *KAR2* overexpression partly restored CR- and CFW-resistance in the *ire1*Δ and *hxl1*Δ mutants, indicating that Kar2 is also involved in the maintenance of cell wall integrity. Interestingly, however, *KAR2* overexpression recovered CR/CFW-resistance more efficiently in the *ire1*Δ mutant than in the *hxl1*Δ mutant (Fig. 4E). Taken together Kar2 has crucial roles in the ER stress response, thermotolerance, and maintenance of cell wall integrity downstream of the Ire1/Hxl1-mediated UPR pathway in *C. neoformans*.

### Kar2 controls genotoxic stress response in an Ire1-dependent, but Hxl1-independent, manner

**Figure 5:**
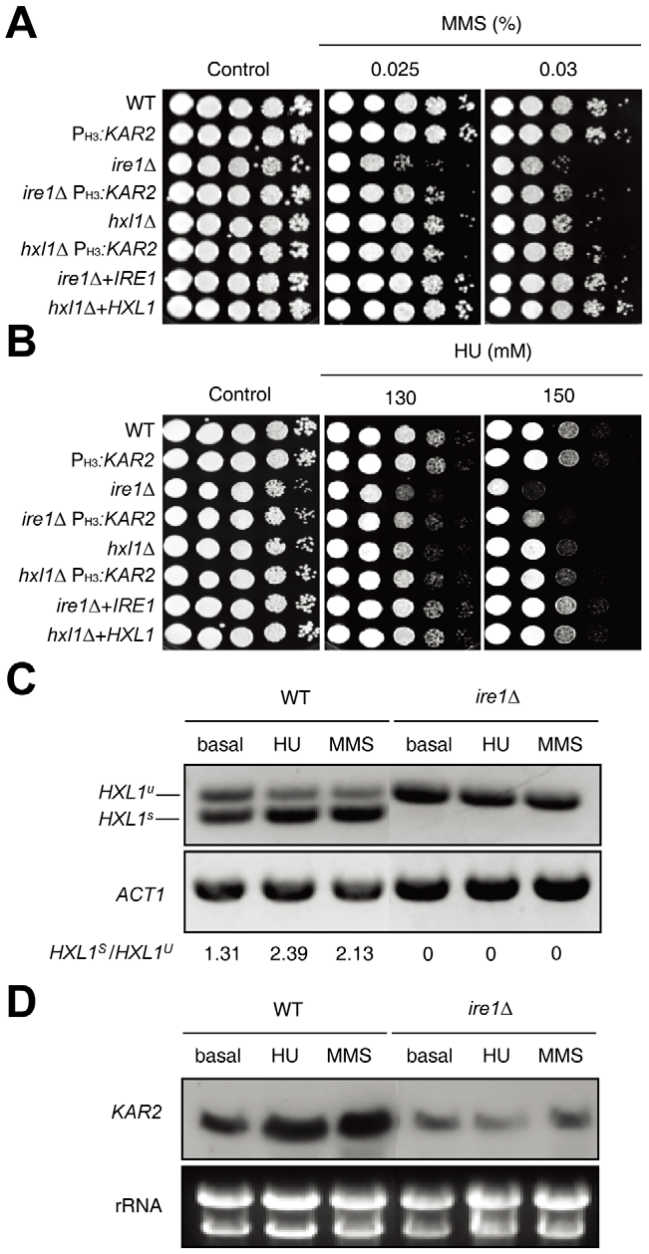
Kar2 mediates genotoxic stress responses downstream of the UPR pathway. (A and B) Cells [the WT H99 strain, *ire1*Δ and *hxl1*Δ mutants, *ire1*Δ+*IRE1* and *hxl1*Δ+*HXL1* complemented strains, and P_H3_ *:KAR2* strains (YSB1751, YSB1741, and YSB1745)] were spotted on a YPD agar medium containing the indicated concentrations of DNA damage inducers, including methyl methanesulfonate (MMS; A) or hydroxyl urea (HU; B), incubated at 30^o^C for 2–4 days, and photographed. (C) The RT-PCR analysis of UPR-induced *HXL1* splicing was performed with cDNA samples prepared from total RNA samples of the WT H99 strain and *ire1*Δ mutants treated with HU (90 mM) or MMS (0.03%) for 1 h. (D) Using the total RNA set from (C), Northern blot assay was performed to measure *KAR2* induction levels in the WT H99 strain and *ire1*Δ mutants. The Northern blot membrane was hybridized with the *KAR2* specific probe, washed, and developed.

Genotoxic stress is likely to cause ER stress indirectly because DNA damage leads to production of truncated or mutated proteins, which could accumulate as misfolded or unfolded toxic proteins in the ER. Supporting this idea, the *ire1*Δ and *hxl1*Δ mutants were hypersensitive to methyl methanesulfonate (MMS), which is a DNA alkylating agent that causes DNA mutagenesis by base mispairing and replication blocking [19], compared to WT (Fig. 5A). Verifying the result, reintegration of *IRE1* or *HXL1* restored the WT levels of MMS- resistance (Fig. 5A). Overexpression of *KAR2* partly restored MMS-resistance in the *ire1*Δ mutant, but not in the *hxl1*Δ mutant, suggesting that Kar2 is involved in the genotoxic stress response downstream of Ire1 (Fig. 5A). Similarly, the *ire1*Δ mutant exhibited a greater sensitivity to hydroxyurea (HU), which inhibits DNA replication by blocking ribonucleotide reductase [20], than WT whereas the *hxl1*Δ mutant showed only a slightly increased sensitivity to HU (Fig. 5B). *KAR2* overexpression partly recovered the HU-resistance in the *ire1*Δ and *hxl1*Δ mutants (Fig. 5B).

To test the hypothesis that genotoxic stress causes ER stress, which could activate UPR pathway and induces *KAR2* expression, we performed RT-analysis of *HXL1* splicing and Northern blot assay to monitor *KAR2* induction treated with the two DNA damaging agents, MMS and HU. Interestingly, the unconventional splicing event in the *HXL1* mRNA was enhanced in WT when treated with HU or MMS (Fig. 5C). Furthermore, the treatment of HU or MMS induced *KAR2* expression in WT strain, but not in the *ire1*Δ (Fig. 5D), indicating that the HU or MMS treatment activates the *KAR2* induction in the Ire1/Hxl1-dependent manner.

Therefore, genotoxic stress causes ER stress and activates the UPR pathway, partly through the Kar2-dependent manner.

### Kar2 controls azole drug susceptibility downstream of the UPR pathway in Erg11- and Erg3-independent manners in *C. neoformans*

**Figure 6:**
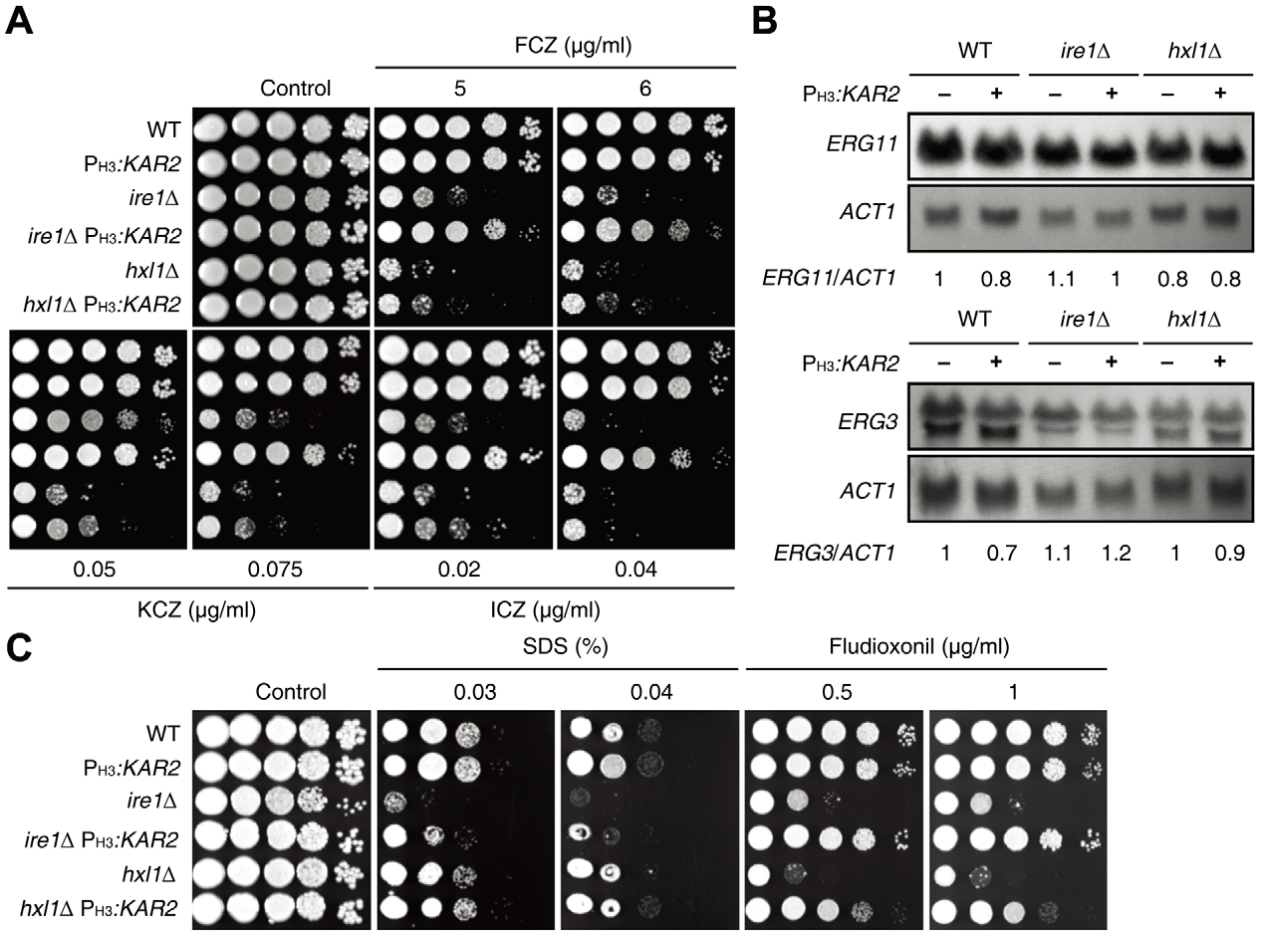
Kar2 has a role in azole drug resistance downstream of the UPR pathway without affecting *ERG11* and *ERG3* expression. (A) WT H99 strain, *ire1*Δ and *hxl1*Δ mutants, and P_H3_ *:KAR2* strains (YSB1751, YSB1741, and YSB1745) were grown for 16 hr at 30^o^C in a liquid YPD medium, 10-fold serially diluted and spotted on a YPD agar medium containing the indicated concentrations of azole drugs and photographed. (B) The expression levels of *ERG11* and *ERG3* in strains described in (A). Each membrane was hybridized with an *ERG11* or *ERG3-*specific probe, washed, and developed. Subsequently, the same membrane was stripped, subjected to re-hybridization with the *ACT1*-specific probe, washed, and developed. Expression levels of *ERG11* or *ERG3* were quantitatively measured with a phosphorimager and normalized with those of *ACT1*. Each *KAR2*/*ACT1* is a value relative to that of the WT strain set to 1.0. (C) Strains described in (A) were grown for 16 hr at 30°C in a liquid YPD medium, 10-fold serially diluted, spotted on a YPD agar medium containing the indicated concentrations of SDS and fludioxonil, and photographed after incubation for 3 days.

Ergosterol is the major sterol found in the membrane of fungi and has critical roles in controlling membrane stability and fluidity [21]. Since ergosterol is replaced by cholesterol in the human cell membrane, ergosterol or its synthesis pathway has been exploited as the major target for modulating fungal infection. For example, a polyene class of amphotericin B binds to ergosterol and produces pores in the fungal membrane, which lead to a lack of ions such as potassium. The azole class of drugs directly inhibits ergosterol synthesis from lanosterol at different steps [22]–[24]. Since the ER is the place where sterol biosynthesis occurs, the UPR pathway could be related to ergosterol biosynthesis and antifungal drug resistance. In fact, UPR signaling mutants are significantly susceptible to azole drugs [6], [7], [9]. In *C. neoformans*, both *ire1*Δ and *hxl1*Δ mutants are hypersensitive to azole drugs, such as fluconazole, ketoconazole, and itraconazole [7] (Fig. 6A), indicating that Ire1 and Hxl1 may have redundant and discrete roles in controlling azole resistance. Here, we determined whether Kar2 is involved in azole resistance downstream of the UPR pathway. Surprisingly, the overexpression of *KAR2* partly but significantly restored the azole resistance of the *ire1*Δ mutant, but only slightly restored the azole resistance of the *hxl1*Δ mutant (Fig. 6A). Similar to the cell wall stress response, *KAR2* overexpression recovered the azole drug resistance more efficiently in the *ire1*Δ mutant than in the *hxl1*Δ mutant at low levels of the azole drug. These data further support the hypothesis that Ire1 and Hxl1 control azole resistance in different ways and that the role of Ire1 in azole resistance mainly depends on Kar2.

Transcriptome analysis in *A. fumigatus* revealed that the expression levels of ergosterol biosynthesis genes, such as *ERG11* and *ERG3*, in *ire1*Δ and *hac1*Δ mutants are lower than those in WT [14]. This finding led us to examine the expression patterns of the ergosterol synthesis genes in the WT strain and *ire1*Δ and *hxl1*Δ mutants with or without *KAR2* overexpression by Northern blot analysis in *C. neoformans*. In stark contrast to the results in *A. fumigatus*, the expression levels of *ERG11* and *ERG3* were not significantly affected by the *ire1*Δ and *hxl1*Δ mutations in *C. neoformans* (Fig. 6B). Furthermore, *KAR2* overexpression did not significantly change the expression levels of *ERG11* and *ERG3* (Fig. 6B). All of these data suggest that the role of the UPR pathway in azole resistance is mainly independent of the regulation of *ERG11* or *ERG3* expression in *C. neoformans*.

Given the above, it remains to be answered how the UPR pathway is involved in azole resistance, partly through the Kar2 molecular chaperone, in *C. neoformans.* Accumulation of toxic sterol intermediates by treatment with azole disrupts the membrane integrity [25], [26]. Therefore, there is a possibility that mutation of the UPR pathway may disrupt membrane stability, which may synergize with azole treatment for antifungal activity. Supporting this idea, there is a report that Hsp90, a molecular chaperone, suppresses azole susceptibility of cells by stabilizing a catalytic subunit of calcineurin that is required for azole tolerance [25]. To address this possibility, we tested the membrane stability of the UPR mutants by using SDS (sodium dodecyl sulfate), which is an ionic detergent, disrupting cell membrane stability. The *ire1Δ* mutant exhibited increased sensitivity to SDS and overexpression of *KAR2* restored the SDS-resistance in the *ire1*Δ mutant (Fig. 6C). Interestingly, however, the *hxl1*Δ mutant was almost as susceptible or only slightly more susceptible to SDS as that of WT. We tested another membrane destabilizer, fludioxonil. Fludioxonil is a phenylpyrrole antifungal drug and hyperactivates Hog1 MAP kinase (MAPK) to induce over-accumulation of intracellular glycerol, which increases intracellular turgor pressure and indirectly affects cell membrane stability, resulting in defective cytokinesis and cell swelling [27]. *KAR2* overexpression suppressed the increased fludioxonil susceptibility in both the *ire1Δ* and *hxl1Δ* mutants. Interestingly, similar to the azole drug test, *KAR2* overexpression rescued fludioxonil-resistance more efficiently in the *ire1Δ* mutant than in the *hxl1*Δ mutant (Fig. 6C).

Taken together, the UPR pathway appears to be involved in azole resistance by controlling membrane stability, partly through the Kar2 molecular chaperone, but not by affecting the expression of ergosterol biosynthesis genes, such as *ERG11* and *ERG3*, in *C. neoformans*.

### Azole treatment is sensed as ER stress and activates the UPR pathway and induction of *KAR2* in *C. neoformans*

**Figure 7:**
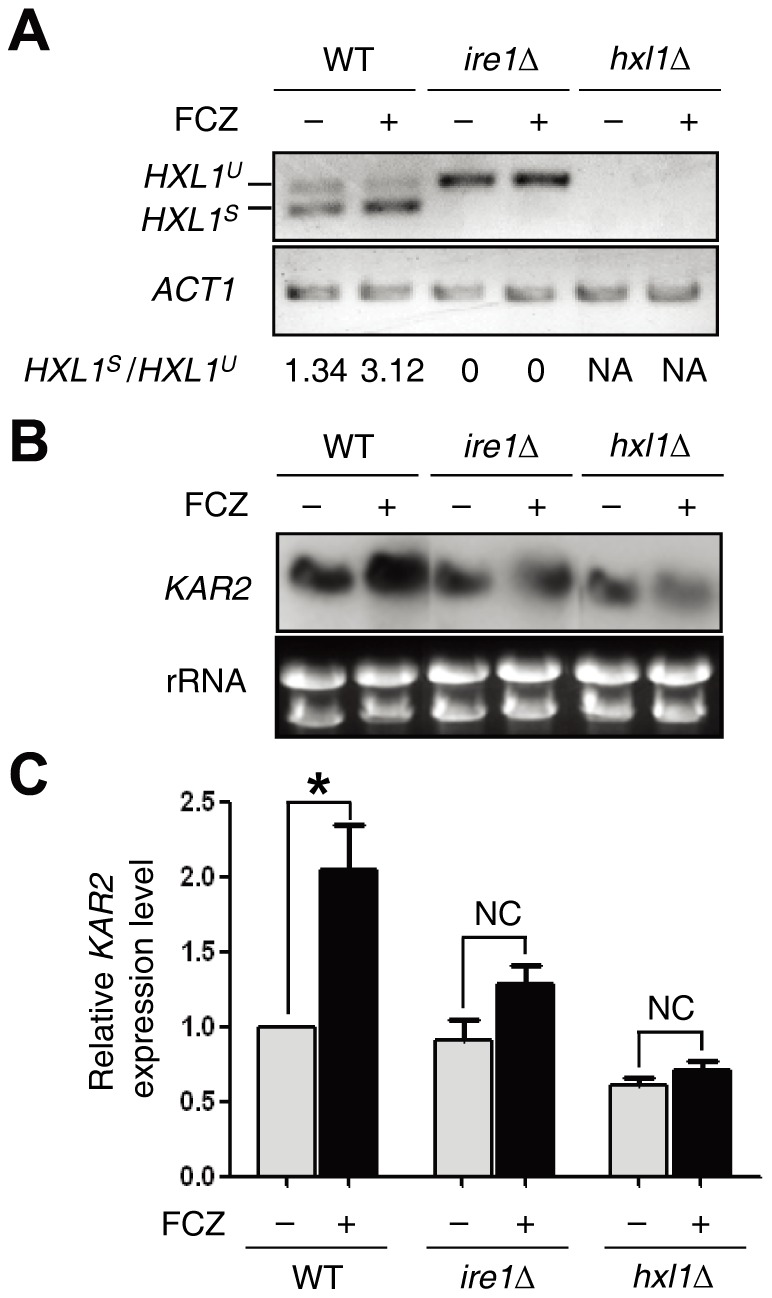
Azole treatment induces the *HXL1* unconventional splicing event in the UPR pathway and upregulation of *KAR2* in *C. neoformans*. (A) The RT-PCR analysis of UPR-induced *HXL1* splicing was performed with cDNA samples prepared from total RNA samples of the WT H99 strain and *ire1*Δ and *hxl1*Δ mutants treated with or without fluconazole (FCZ, 10 μg/ml) for 1h. NA, not available. (B and C) Using the same total RNA set of (A), Northern blot assay and qRT-PCR analysis were performed to monitor *KAR2* induction levels. For quantitative qRT-PCR analysis, *KAR2* expression levels were normalized with *ACT1* as a control. Relative *KAR2* expression levels indicate the ratio of the normalized *KAR2* expression level of each strain with or without FCZ (10 µg/ml) to that of WT H99 strain at zero time point without FCZ. RT-PCR of *HXL1* and *ACT1*, qRT-PCR analysis, and Northern blot analysis were performed with gene-specific primers or probes as described in the Materials and Methods.

As an additional explanation for the role of the UPR pathway in azole susceptibility, it is possible that azole drugs may cause ER stress, which could activate the UPR pathway and induction of *KAR2* to counteract their effects. To test this hypothesis, we examined whether azole treatment could activate the UPR pathway. As a first hallmark for the UPR activation, the Ire1-mediated Hxl1 splicing event was monitored in cells treated with fluconazole (Fig. 7A). Surprisingly, the unconventional splicing event in the *HXL1* mRNA significantly increased upon fluconazole treatment (Fig. 7A). Because such an *HXL1* splicing event was not present in the *ire1*Δ mutant, the azole-mediated *HXL1* mRNA splicing clearly depends on the Ire1 kinase (Fig. 7A).

As a second hallmark for the UPR activation, we examined whether the azole treatment could induce the expression of *KAR2*. Northern blot analysis revealed that the treatment of fluconazole slightly induced *KAR2* expression in WT strain, but not in the *ire1*Δ or *hxl1*Δ mutant (Fig. 7B). This result was further confirmed by quantitative reverse transcription-PCR (qRT-PCR) analysis (Fig. 7C), indicating that the azole treatment activates the *KAR2* induction in the Ire1/Hxl1-dependent manner. Taken together, these data suggest that the activation of the UPR pathway is required for counteracting ER stress caused by azole treatment in *C. neoformans*.

### *KAR2* is not involved in Ire1-mediated capsule regulation in *C. neoformans*

**Figure 8:**
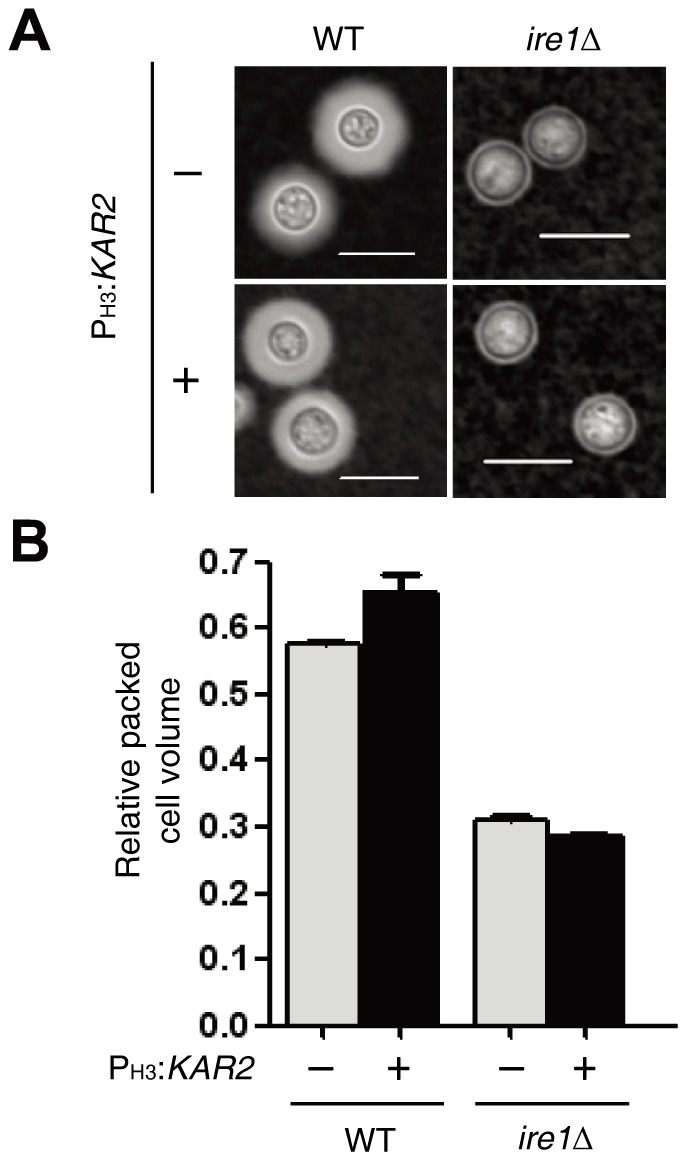
Kar2 is not sufficient for Ire1-mediated regulation of capsule production in *C. neoformans*. To measure the capsule production, each strain [WT H99 strain, the *ire1Δ* mutant (YSB552), and P_H3_ *:KAR2* strains (YSB1741 and YSB1751)] was spotted and cultured on a DME agar medium at 30^o^C for 2 days. Capsules were visualized by India ink staining (A), and the relative capsule volume was measured by calculating the ratio of the length of the packed cell volume phase per length of the total volume phase (B). Three independent experiments with technical triplicates were performed. The scale bar represents 10 µm. Error bars represent the standard deviation.

The capsule is one of the key virulence factors in *C. neoformans* because it prevents cells from being phagocytized by macrophages. Capsule production is regulated by the iron concentration and physiological CO_2_ levels [28]. A polysaccharide capsule is secreted by Sec4 or Sec6-mediated exocytosis into the extracellular space [29], [30]. Our previous study reported that the *ire1*Δ mutant, but not the *hxl1*Δ mutant, is highly defective in capsule production [7]. To address whether Kar2 is involved in Ire1-mediated capsule biosynthesis, we compared the capsule production levels between the *ire1*Δ mutant and the *ire1*Δ P*_H3_:KAR2* strain. Overexpression of *KAR2* did not rescue capsule defects in the *ire1*Δ mutant in both qualitative (Fig. 8A) and quantitative measurement (Fig. 8B). This indicates that Ire1 in the UPR pathway is involved in capsule production in an Hxl1- and Kar2-independnet manner or *KAR2* overexpression is not sufficient to restore the capsule production in the *ire1Δ* mutant without concomitant expression of other factors.

## Discussion

Cellular functions of the ER-resident molecular chaperone BiP/Kar2, and its connection to the UPR signaling pathway, have been characterized in models including budding yeast and animal cells, but not in basidiomycete fungi. In this study, we for the first time functionally characterized a Kar2/BiP protein in the UPR pathway in a basidiomycete fungus, *C. neoformans*. Our previous study revealed that expression of *KAR2* is induced by the evolutionarily conserved Ire1 kinase and a unique Hxl1 transcription factor under ER stress induced by TM or a temperature upshift in *C. neoformans* [7]. During preparation of this manuscript, the function of Kar2 in sexual differentiation of *C. neoformans* was reported [31]. However, the roles of Kar2 in the UPR pathway as a molecular chaperone remain unexplored in *C. neoformans*.

The requirement of Kar2 for cell survival has been investigated in opportunistic fungal pathogens as well as budding yeast [10], [11], [31]. Our study also demonstrated that Kar2 is essential for the viability of *C. neoformans*, as it is for other fungi. Conditional null *kar2* mutant strains exhibited severe growth defects under repressed conditions (Fig. 2C). This result is in agreement with a previous study proposing that Kar2 is essential for the viability of *C. neoformans* [31]. Recent studies have reported that Kar2 has a wide range of roles including translocation, protein folding, and nuclear fusion [10]–[14]. Protein translocation, especially, is essential for localization of secreted or folded proteins to their correct sites during the secretory process. Impairment of protein folding and secretion leads to an accumulation of toxic proteins, which results in cell death. Therefore, Kar2 is likely to be involved in key cellular processes for the survival of eukaryotes.

In this study, several lines of evidence demonstrated that Kar2 acts as one of the downstream effectors of the UPR signaling pathway to counteract ER stress, high temperature stress, and cell wall destabilizing stress. First, the overexpression of *KAR2* by the H3 promoter partly restored resistance to ER stress, high temperature, and cell wall destabilizing stress in the *ire1*Δ and *hxl1*Δ mutants (Fig. 4). Interestingly, however, overexpression of *KAR2* recovered cell wall stress resistance in the *ire1*Δ mutant more efficiently than in the *hxl1Δ* mutant, further supporting the idea that Ire1 and Hxl1 do not strictly have a linear relationship in the UPR pathway. Second, the abundance of *KAR2* mRNA is controlled by Ire1 kinase and Hxl1 transcription factor [7]. *KAR2* expression is induced by ER stress or a temperature upshift in both Ire1- and Hxl1-dependent manners [7] (Fig. 3C). In conclusion, Kar2 operates downstream of the Ire1 and Hxl1 in the UPR pathway to control ER stress, high temperature growth, and maintenance of cell wall integrity.

The finding that *KAR2* overexpression restores DTT resistance more efficiently than TM resistance in the *ire1*Δ and *hxl1*Δ mutants implies that Kar2 is better suited as a molecular chaperone for resolving unfolded proteins generated by the perturbed redox state of the ER rather than by a lack of *N-*glycosylation-dependent folding capacity. In the ER, non-glycoproteins undergo proper folding through protein disulfide isomerase (PDI) and Kar2/BiP, whereas glycoproteins undergo *N*-glycan-dependent protein folding mediated by PDI and the Calnexin cycle [32]. Given that the ER has an oxidized environment that assists in efficient disulfide bond formation for protein folding [33], treatment with a reducing agent, such as DTT, significantly affects normal protein folding in the ER. Therefore, it is conceivable that accumulated misfolded non-glycoproteins by DTT treatment could be efficiently resolved by *KAR2* overexpression. In contrast, TM inhibits the first step of *N*-linked glycosylation by blocking the transfer of the *N*-acetylglucosamine-1-phosphate (GlcNAc-1-P) group of UDP-GlcNAc to dolichol-p, which subsequently blocks PDI/Calnexin-mediated *N-*glycan-dependent protein folding and causes the accumulation of unfolded proteins [34]. Therefore, *KAR2* overexpression may have only a limited role in resolving the TM-mediated protein misfolding unless proper *N-*linked glycosylation is provided. Similar to DTT, treatment with diamide, a diazine compound inducing the formation of disulfide bonds, may also generate unnaturally folded proteins in the ER, which could be efficiently resolved by *KAR2* overexpression. In summary, our data indicate that the Kar2 molecular chaperone is more suitable for dealing with misfolded or unfolded proteins resulting from a change in the redox state of the ER rather than for the quality control of glycoproteins in the ER.

One of the notable findings in this study is that the UPR pathway controls the genotoxic stress response partly through Kar2. It is not surprising that the UPR pathway is involved in defense against genotoxic stress because DNA damage leads to the production of truncated or mutated proteins, which could accumulate as misfolded or unfolded toxic proteins in the ER. In fact, the involvement of the UPR pathway in the genotoxic stress response has been reported in other organisms [35]. It is possible that Kar2 proteins prevent misfolded proteins caused by genotoxic stress from aggregating by acting as a molecular chaperone. In *S. cerevisiae,* strains having defects in *KAR2* induction become greatly sensitive to increased expression of mutated carboxypeptidase Y (CPY^*^), which is widely used as a model misfolded protein [36]. It was not unexpected to find that Kar2 overexpression only partly suppressed genotoxic sensitivity in the *ire1*Δ mutant because Ire1 must have other downstream effector(s), other than Kar2, to counteract genotoxic stresses. Furthermore the fact that the *ire1*Δ mutant is much more susceptible to genotoxic agents than the *hxl1*Δ mutant indicates that Ire1 may control genotoxic stress response in both Hxl1-dependent and independent manners. These data provide further support for the idea that Ire1 has both Hxl1-independent and dependent roles in *C. neoformans.*

The striking role of the UPR pathway in azole susceptibility is of clinical importance, but its mode of action remains puzzling. Previously, we have shown that the inhibition of the UPR pathway greatly increases azole susceptibility in *C. neoformans*, suggesting that the signaling components of the UPR pathway, such as Ire1 and Hxl1, could be excellent antifungal drug targets for combination therapy with azole drugs [7]. Particularly because Hxl1 is a unique transcription factor, which is phylogenetically distinct from the Xbp1 transcription factor in humans, it is an attractive antifungal drug target. Askew and colleagues reported a similar finding in *A. fumigatus*. The *A. fumigatus* strain with a *hacA* gene deletion, which encodes a yeast Hac1 ortholog, is more susceptible to azoles (e.g. ICZ and FCZ) and polyene (e.g. amphotericin B, AMB) than the WT strain [9]. More recently, they proposed a potential mechanism explaining how the UPR pathway mutants are involved in azole susceptibility [6]. Feng et al. performed DNA microarray analysis and found that the expression of *ERG11*, encoding a lanosterol 14α-demethylase that is a target of most azole drugs, decreases in the *hacA* and *ireA* mutants. Supporting this finding, cellular ergosterol levels were also shown to decrease in the UPR mutants [6]. The explanation remains elusive, however, for why the *hacA* mutant is highly susceptible to AMB in *A. fumigatus* [9] given that the decreased ergosterol content could confer resistance to AMB, which binds ergosterol. Probably multiple reasons exist for the increased susceptibility of the UPR mutants to both azole and polyene drugs in *A. fumigatus*. Interestingly, the *hac1Δ* mutant in *S. cerevisiae* is as resistant to azole drugs as the wild-type. Therefore, the role and regulatory mechanisms of the UPR pathway in azole resistance appears to be highly divergent between fungal species.

This study provides a potential mechanism for the role of the UPR pathway in azole susceptibility in *C. neoformans*. Under unstressed condition, the UPR pathway is not involved in ergosterol biosynthesis, given the fact that the expression levels of *ERG11* and *ERG3* are not significantly affected by mutations in *IRE1* or *HXL1* (Fig. 6B). Supporting this, the UPR mutants of *C. neoformans* that are highly susceptible to most azole drugs are also susceptible to AMB [7]. Treatment with azole drugs, however, may confer an ER stress to cells, which the UPR pathway must be activated to counteract. Supporting this, it was discovered that the Ire1-mediated Hxl1 splicing increases in response to FCZ treatment (Fig. 7). Furthermore, *KAR2* expression was found to be induced by FCZ treatment in the Ire1/Hxl1-dependent manner. These findings may explain why *KAR2* overexpression considerably recovers normal azole resistance in the *ire1Δ* mutant without affecting the *ERG11* and *ERG3* expression levels. Nevertheless, Ire1 and Hxl1 appear to differentially control azole resistance in *C. neoformans* because restoration of azole resistance by *KAR2* overexpression is not as efficient in the *hxl1Δ* mutant as in the *ire1*Δ mutant. In addition to this defensive role of the UPR pathway in azole treatment, the requirement of the UPR pathway for maintaining membrane stability may also contribute to the synergism with azole treatment for antifungal activity.

Currently the nature of the ER stress by azole treatment still remains elusive. In eukaryotes, sterols are synthesized in the ER and then transported to the plasma membrane (PM) mainly independent of a classical secretory pathway. Sterols are highly enriched in the PM but their concentration is low in the ER membrane [37]. Although it is not clear how the inhibition of sterol biosynthesis by azole drugs activates the UPR pathway and Kar2/BiP induction, accumulation of ER membrane cholesterol induces ER stress and apoptosis in mammals and the UPR pathway regulates expression of genes involved in lipid metabolism in *S. cerevisiae* [5], [38], [39]. Furthermore, it has been reported that disruption of ER function induces lipid dysregulation [40] and *vice versa*, lipotoxicity triggered by lipid imbalance leads ER stress, which activates the UPR pathway [41]. Therefore, it is conceivable that perturbation of sterol/lipid metabolism by azole drug treatment may cause ER stress and activate the UPR pathway in *C. neoformans*. Functional correlation between the UPR and sterol/lipid metabolic pathways needs to be further investigated in future studies.

Thus far, most of the Ire1-dependent phenotypes, including the ER stress response, thermotolerance, maintenance of cell wall/membrane integrity, genotoxic stress response, and antifungal drug resistance, have been found at least partly to depend on the functions of the Kar2 molecular chaperone, except in the case of capsule production. *KAR2* overexpression did not restore capsule production defects in the *ire1*Δ mutants at all. Therefore, the role of Ire1 in capsule synthesis appears to be independent of Hxl1 and Kar2. In fact, Ire1 may not directly affect capsule biosynthesis *per se*, but control the secretion of polysaccharide capsular precursors onto the cell surface. In yeast, it is known that the UPR pathway is involved in the secretory pathway. Our previous study also showed that some of the secretion-related genes, including *SEC61,* are regulated by the UPR pathway [7]. However, the exact regulatory mechanism of Ire1 in capsule production remains to be elucidated further in future studies.

In conclusion, the molecular chaperone Kar2/BiP has pleiotropic roles in cell viability, ER stress response, thermotolerance, maintenance of cell wall/membrane integrity, genotoxic stress response, and azole drug resistance, but not in capsule production, downstream of the Ire1/Hxl1-dependent UPR signaling pathway in *C. neoformans.*

## Materials and Methods

### Strains and growth conditions

*C. neoformans* strains used in the study are listed in Table 1 and were cultured on a yeast extract-peptone-dextrose (YPD) medium. For capsule production assay, the agar-based Dulbecco Modified Eagle (DME, Invitrogen, Carlsbad, CA) medium was used [42], [43].

**Table 1:** Strains used in this study.

Strain	Genotype	Parent	Reference
***C. neoformans***			
H99	*MAT*α		[49]
YSB552	*MAT*α *ire1*Δ*::NAT-STM#224*	H99	[7]
YSB723	*MAT*α *hxl1*Δ*::NAT-STM#295*	H99	[7]
YSB1000	*MAT*α *ire1*Δ*::NAT-STM#224 IRE1-NEO*	YSB552	[7]
YSB762	*MAT*α *hxl1*Δ*::NAT-STM#295 HXL1-NEO*	YSB723	[7]
YSB1637	*MAT*α *P_CTR4_:KAR2 NAT*	H99	This study
YSB1638	*MAT*α *P_CTR4_:KAR2 NAT*	H99	This study
YSB1639	*MAT*α *P_CTR4_:KAR2 NAT*	H99	This study
YSB1640	*MAT*α *P_CTR4_:KAR2 NAT*	H99	This study
YSB1741	*MAT*α *ire1*Δ*::NAT-STM#224 P*_H3_ *:KAR2 NEO*	YSB552	This study
YSB1744	*MAT*α *ire1*Δ*::NAT-STM#224 P*_H3_ *:KAR2 NEO*	YSB552	This study
YSB1745	*MAT*α *hxl1*Δ*::NAT-STM#295 P*_H3_ *:KAR2 NEO*	YSB723	This study
YSB1746	*MAT*α *hxl1*Δ*::NAT-STM#295 P*_H3_ *:KAR2 NEO*	YSB723	This study
YSB1751	*MAT*α *P*_H3_ *:KAR2 NEO*	H99	This study
YSB1752	*MAT*α *P*_H3_ *:KAR2 NEO*	H99	This study

Each *NAT-STM#* indicates the Nat^r^ marker with a unique signature tag.

### Rapid amplification of cDNA ends (RACE) analysis of 5′ and 3′ untranslated regions (UTRs) of the *KAR2* gene

To characterize 5′ and 3′ untranslated regions (UTRs) and coding sequence of the *KAR2* genes, we performed rapid amplification of cDNA ends (RACE) analysis with GeneRacer Kit (Invitrogen, Carlsbad, CA). The total RNA of the WT H99 strain incubated overnight at 30°C was isolated with RiboEX (GeneAll, Korea) according to the manufacturer's instructions. Each 5′ and 3′ RACE products of *KAR2* were cloned into the pTOP-V2 (Enzynomics) and sequenced. The 5′ and 3′ UTR and coding sequences of *KAR2* have been deposited in GenBank (accession number JX982102).

### Construction of the P*_CTR4_:KAR2* and P*_H3_:KAR2* strains

To replace the native *KAR2* promoter with the copper-regulated *CTR4* promoter, we generated a *KAR2* promoter replacement cassette as follows. The 5′-flanking region of *KAR2* and the 3′-flanking region of *KAR2* were amplified for homologous recombination by PCR with primer pairs B3551 (5′-CGTAGGGTATGTCTCTGATGAG-3′)/B3650 (5′-CACTCGAATCCTGCATGCAAAGTCTTGAGGAATAGACAA-3′) and B3651 (5′-CGACAACGACTTCACCAATCTGCCACCATGGCATACCCT-3′)/B3652 (5′-ACTCCTGTTTGCCACTTCG-3′), respectively. B354 (5′-GCATGCAGGATTCGAGTG-3′) and B355 (5′-GATTGGTGAAGTCGTTGTCG-3′) primers were used for PCR-amplification of the *NAT*-*CTR4* promoter using pNAT-CTR4-2 as a template [18]. The *KAR2* promoter replacement cassette was produced by double joint PCR with primer pairs, B3551/B1455 (5′-AACTCCGTCGCGAGCCCCATCAAC-3′) for the 5′-franking region and B3652/B1454 (5′-AAGGTGTTCCCCGACGACGAATCG-3′) for the 3′-franking region, and the WT H99 strain was biolistically transformed, as previously described [44], [45]. The correct insertion was confirmed with Southern blot analysis, as previously described [46].

To replace the native *KAR2* promoter with the histone H3 promoter, the P*_H3_:KAR2* cassette was constructed as follows. Primers B3551 and B4270 (5′-CACTCGAATCCTGCATGCGGTGGCAAAAGTCTTGAGGA-3′) for the 5′-flanking region of the *KAR2* gene and primers B4264 (5′-CAAGACCTCAAAGACACCG-3′) and B4271 (5′-ACCACAACACATCTATCACATGGCATACCCTTCAAGAAT-3′) for the exon of the *KAR2* gene were used in the first round PCR. The 5′- and 3′-regions of the dominant selectable *NEO* marker (neomycin/G418-resistant marker) were amplified with primer pairs B4017 (5′-GCATGCAGGATTCGAGTG-3′)/B1887 (5′-ATTGTCTGTTGTGCCCAG-3′) and B4018 (5′-GTGATAGATGTGTTGTGGTG-3′)/B1886 (5′-TGGAAGAGATGGATGTGC-3′), respectively. Next, the 5′ and 3′ regions of the *NEO*-marked histone H3 promoter cassette were amplified by double joint PCR with primer pairs B3551/B1887 and B4271/B1886, respectively. The P*_H3_:KAR2* strains were generated by introducing the *NEO*-marked H3 promoter cassette into *C. neoformans* serotype A H99 strain and *ire1*Δ and *hxl1*Δ mutants by biolistic transformation, as previously described [44], [45]. Stable transformants selected on the YPD medium containing G418 were screened by diagnostic PCR with primers B3550 (5′-TCCCAATCTACTGACCTATCG-3′) and B79 (5′-TGTGGATGCTGGCGGAGGATA-3′). Next, the correct genotypes were verified by Southern blot analysis, as previously described [46]. A probe for the *KAR2* gene was amplified with primers B3551 and B3555 (5′- CAAGCAGGGACAGTAACAAC-3′).

### Total RNA isolation, Northern blot assay, and qRT-PCR analysis

To evaluate *KAR2* expression levels and patterns, total RNA isolation and Northern blot or qRT-PCR were performed as follows. Strains were cultured in a 30 ml YPD liquid medium for 16 hr at 30°C. Then 5 ml of overnight culture was inoculated into 50 ml of fresh YPD medium, further incubated at 30°C until the optical density at 600 nm (OD_600_) of the culture medium reached approximately 1.0, and then pelleted by centrifugation for total RNA isolation. To monitor expression levels of *KAR2* in WT and P*_H3_:KAR2* strains in response to TM, the overnight culture was inoculated into 150 ml of fresh YPD medium and incubated at 30°C up to an OD_600_ of 1.0. For the zero time sample, 50 ml out of the 150 ml culture was sampled and pelleted by centrifugation. The remaining 100 ml culture was treated with the indicated concentrations of TM. During incubation, a 50 ml culture was sampled at 30 and 60 min. Total RNA was isolated by the TRIzol reagent (RiboEx) as previously described [47]. Northern blot analysis was performed with 10 μg of total RNA from each strain. Electrophoresis, membrane transfer, hybridization, and washing were performed by following the protocol previously described [46]. A probe for the *KAR2* gene was amplified with primers B4978 (5′- AGGCAGTCTGGAGTGTCATC-3′) and B3652. The qRT-PCR for quantitatively measuring relative expression level of *KAR2* was performed with gene specific primers B679 (5′-CGCCCTTGCTCCTTCTTCTATG-3′)/B680 (5′-GACTCGTCGTATTCGCTCTTCG-3′) for *ACT1*, as a reference, and B5253 (5′-CTCTGAGGACGACAAGGACA-3′)/B5254 (5′-AGCTCAGAAAGCTGCTCCTC-3′) for *KAR2*.

### Stress sensitivity test

Each strain was incubated overnight (about 16 hr) at 30°C in a liquid YPD medium, washed, serially diluted (1 to 10^4^ dilutions) with dH_2_O, and spotted (3 µl) onto a solid YPD medium containing the indicated concentration of stress inducers. To test ER stress and cell wall stress, cells were spotted onto a solid YPD medium containing the indicated concentration of ER stress inducers, such as tunicamycin (TM, Sigma) or dithiothreitol (DTT, Sigma), cell wall stress inducers, such as calcofluor white (CFW, Sigma) or congo red (CR, Sigma), or cell membrane destabilizers, such as sodium dodecyl sulfate (SDS, Sigma) and fludioxonil (Sigma). To examine antifungal drug resistance, azole drugs including fluconazole, ketoconazole, and itraconazole were used. For genotoxic DNA damage stress, cells were spotted onto a solid YPD medium containing 130 and 150 mM hydroxyurea (HU, Sigma) and 0.02, 0.025, and 0.03% methylmethan sulfonate (MMS, Sigma). To test thermosensitivity, cells were incubated at 30, 35, 37, and 39°C and photographed after 2 to 3 days.

### Monitoring *HXL1* splicing event

To monitor splicing levels of *HXL1* in WT and P*_H3_:KAR2* strains, samples were prepared as follows. Strains were cultured in a 50 ml YPD liquid medium for 16 hr at 30°C. Then the overnight culture was inoculated at 1:20 dilution into a fresh 150 ml YPD medium and incubated at 30°C until the OD_600_ of the culture medium reached approximately 1.0. For the zero time sample, 50 ml out of the 150 ml culture was sampled. The remaining 100 ml culture was treated with indicated concentrations of TM, DTT, fluconazole, MMS, and HU. During incubation, a 50 ml culture was sampled at 30 and/or 60 min. Total RNAs were isolated with the TRIzol reagent (RiboEx) as previously described. Single strand cDNA was synthesized using a reverse transcriptase (Fermentas). RT-PCR of *HXL1* and *ACT1* was performed with gene specific primers C19 (5′-CACTCCATTCCTTTCTGC-3′)/C20 (5′- CGTAACTCCACTGTGTCC-3′) and B3294 (5′-GCACCATACCTTCTACAATGAG-3′)/B3295 (5′-ACTTTCGGTGGACGATTG-3′), respectively.

### Capsule test

For the capsule assay, cells were incubated overnight at 30°C in a liquid YPD medium, spotted onto agar-based DME medium, and further incubated for 2 days at 30°C. For quantitative measurement of capsule production, the relative packed cell volume was measured with hematocrit capillary tubes, as previously described [46], [48]. For visualization of capsule production, each cell scraped from the DME medium was resuspended in a phosphate buffer saline (PBS) buffer, and stained with India ink, and visualized by Nikon eclipse Ti microscope.
